# Repeat ablation for persistent atrial fibrillation: what lies beyond lines and veins?

**DOI:** 10.1093/europace/euaf269

**Published:** 2025-10-25

**Authors:** Lucas V A Boersma, Sing-Chien Yap

**Affiliations:** Cardiology Department, St. Antonius Hospital Nieuwegein/Amsterdam UMC, The Netherlands; Cardiology Department, Erasmus MC, Rotterdam, The Netherlands

**This editorial refers to ‘Repeat in situ ablation versus extensive ablation for recurrent persistent atrial fibrillation’, by M. Qin *et al.*, https://doi.org/10.1093/europace/euaf232.**

Catheter ablation has revolutionized the treatment of persistent atrial fibrillation (AF), yet arrhythmia recurrence after a first procedure remains common, with rates approaching >50% at 1 year.^1^ Current guidelines recommend considering repeat ablation, but the optimal strategy for redo procedures is uncertain.^2,3^ In this issue of *Europace*, Qin *et al*.^4^ report a prospective, multicenter, randomized controlled trial directly comparing two strategies for repeat ablation: extra-pulmonary vein (extra-PV) extensive ablation vs. repeat *in situ* ablation focused on previously treated lesion sets.

The trial enrolled 132 patients with recurrent persistent AF after an initial ablation including pulmonary vein isolation (PVI) and linear lesions. Participants were randomized 1:1 to extra-PV extensive ablation (EXT group) or repeat *in situ* ablation (In situ group). In the EXT group, after assessing for PV reconnection, operators performed bi-atrial electrogram mapping and classification using CARTOFINDER software and ablated sites with spatiotemporal dispersion, short-cycle potentials, or focal activity, adding posterior wall isolation and Vein of Marshall ethanol infusion as needed. The In situ group underwent repeat PVI and re-ablation of incompletely blocked linear lesions but no further substrate modification.

At 12 months, freedom from atrial tachyarrhythmia lasting >30 s (the primary endpoint) was significantly higher; 67% of EXT patients compared with 49% of the In-situ group (hazard ratio 0.59, *P* = 0.037). Freedom from AF alone was also significantly higher with extensive ablation (77% vs. 61%, *P* = 0.027). Major adverse event rates were low and similar between groups (1.5% vs. 1.5%).

These findings add important evidence in a field where randomized data are limited. Previous trials of empiric substrate modification during first ablation of persistent AF, such as STAR-AF II and CAPLA, did not consistently improve outcomes.^5,6^ Qin *et al*.’s study^4^ differs by employing individualized, electrogram-guided ablation specifically in the redo setting—where arrhythmogenic drivers may be more complex and less dependent on PV triggers alone. The observed absolute risk reduction of 18% for AF/AT recurrence translates to a number needed to treat of just six patients, a clinically meaningful benefit.

While the study was modest in size (only 123 patients) with a follow-up of only 12 months, the outcomes highlight that individualized substrate-guided ablation can improve rhythm outcomes without compromising safety. The randomized design and rhythm follow-up with multiple 3-day Holter and *trans*-telephonic monitoring during 12-months favored a consistent trial outcome. The main concern of this trial is that the extra-PV extensive ablation protocol was technically complex, requiring advanced mapping, posterior wall isolation, and vein of Marshall ethanol infusion, procedures that require a lot of time and may not be widely reproducible in all centers. Although the CARTOFINDER software was used to guide electrogram interpretation and ablation, the reproducibility of such an individual approach may be challenging. Indeed, the extensive protocol consisted of 7 optional steps, and it is unclear which of these contributed most to the significantly better outcome. Recently, Derval *et al*.^7^ published their MARSHALL plan randomized trial in *de novo* ablation for persistent AF, testing a systematic stepwise approach of ethanol infusion in the Vein of Marshall, followed by PVI, roof line, mitral isthmus line, and cavotricuspid isthmus line, with an optional floor line to create posterior wall isolation. Freedom of atrial arrhythmias was significantly higher, in 88% of Marshall pts compared with 66% in the PVI only group, even without the step of substrate ablation based on arbitrary electrogram characteristics. Recently, the TAILORED-AF trial^8^ tested the benefit of AI-guided electrogram characterization and ablation, which could offer a more objective valuation of the substrate to ablate on top of PVI and linear ablation. But although freedom of AF was higher after 12-months, the freedom of any atrial arrhythmia was not, due to the pro-arrhythmic effects of too much substrate ablation. In the past, advanced systems like Topera, CardioInsight, and Acutus have tried to map and reveal the pathways that were critical to initiate and sustain AF to guide substrate ablation adjunctive to PVI. While initial results were promising, the randomized trials that were needed to establish a consistent meaningful improvement in outcome were negative. New technologies for AI guided ablation such as electrical flow mapping^9^ and vector mapping are emerging, but larger randomized trials are needed to establish their value for specific patient populations.

Most of the extensive ablation protocols suffer from another downside; ablation was performed with radiofrequency energy and the procedures on average required up to 3 h, making the workflow unattractive to serve the ever-growing number of AF patients that are eligible for ablation. The recent surge of pulsed electrical field ablation therefore provides a welcome innovation allowing very rapid PVI leaving more time for substrate modification.^10^ The ADVANTAGE trial^11^ in persistent AF patients showed very rapid PWI on top of PVI, with a 64% freedom of atrial arrhythmias at 12 month. While the workflow with the FARAWAVE catheter was very easy, the outcome was modest, and the study lacked a control arm. Moreover, the FARADISE registry^12^ did not show a better clinical outcome by adding PWI to PVI by PFA. The recent SPHERE-PerAF trial^13^ showed that extensive MARSHALL plan-like linear ablation with a lattice-tip catheter capable of both PFA and RF led to a numerically better outcome (73.5% vs. 66%, *P* = 0.064) than RF ablation with a standard 4-mm catheter, and shortened the procedures by 20 min. This trial also lacked a control arm to understand the added value of the extensive ablation protocol.

The results of Qin *et al*.^4^ challenge the notion that PV reconnection and linear lesion reconduction are the sole drivers of recurrence. Despite re-isolating reconnected veins and re-ablating incomplete lines, the *In situ* group had significantly more recurrences, suggesting that extra-PV sources contribute substantially to arrhythmia maintenance. These findings echo emerging data from recent studies like TAILORED-AF,^8^ FLOW-AF^9^ and ERASE-AF^14^ that support individualized mechanism-based ablation strategies. Maybe such an individualized approach is most appropriate for patients that fail a more systematic Marshall plan ablation. Emerging (large) focal pulsed-field ablation (PFA) technologies will facilitate easier and quicker workflows and hopefully enhance the durability of lesion sets while maintaining an excellent safety profile. For clinicians, the message is clear: for recurrent AF, it may be time to look beyond the pulmonary veins.

## Data Availability

No original data but references to prior studies underlie this editorial review.
